# Organizational factors influencing successful primary care and public health collaboration

**DOI:** 10.1186/s12913-018-3194-7

**Published:** 2018-06-07

**Authors:** Ruta Valaitis, Donna Meagher-Stewart, Ruth Martin-Misener, Sabrina T. Wong, Marjorie MacDonald, Linda O’Mara, The Strengthening Primary Health Care through Primary Care and Public Health Collaboration Team, Andrea Baumann, Paula Brauer, Michael Green, Janusz Kaczorowski, Rachel Savage, Patricia Austin, Kristin MacLellan, Karen McNeil, Nancy Murray, Sandy Isaacs, Leena Chau

**Affiliations:** 1 School of Nursing, McMaster University, HSc Room 3N25, 1280 Maim Street West, Hamilton, ON L8S4K1 Canada; 2 Dalhousie University, Room G26, Forrest Bldg., PO Box 15000, 5869 University Avenue, Halifax, NS B3H 4R2 Canada; 3 UBC School of Nursing and Centre for Health Services and Policy Research, T201 2211 Wesbrook Mall, Vancouver, BC V6T 2B5 Canada; 4 University of Victoria, HSD B220, 3800 Finnerty Road, Victoria, BC V8P 5C2 Canada

**Keywords:** Primary care, Public health, Organization, Collaboration, Partnership, Health care sector

## Abstract

**Background:**

Public health and primary care are distinct sectors within western health care systems. Within each sector, work is carried out in the context of organizations, for example, public health units and primary care clinics. Building on a scoping literature review, our study aimed to identify the influencing factors within these organizations that affect the ability of these health care sectors to collaborate with one another in the Canadian context. Relationships between these factors were also explored.

**Methods:**

We conducted an interpretive descriptive qualitative study involving in-depth interviews with 74 key informants from three provinces, one each in western, central and eastern Canada, and others representing national organizations, government, or associations. The sample included policy makers, managers, and direct service providers in public health and primary care.

**Results:**

Seven major organizational influencing factors on collaboration were identified: 1) Clear Mandates, Vision, and Goals; 2) Strategic Coordination and Communication Mechanisms between Partners; 3) Formal Organizational Leaders as Collaborative Champions; 4) Collaborative Organizational Culture; 5) Optimal Use of Resources; 6) Optimal Use of Human Resources; and 7) Collaborative Approaches to Programs and Services Delivery.

**Conclusion:**

While each influencing factor was distinct, the many interactions among these influences are indicative of the complex nature of public health and primary care collaboration. These results can be useful for those working to set up new or maintain existing collaborations with public health and primary care which may or may not include other organizations.

**Electronic supplementary material:**

The online version of this article (10.1186/s12913-018-3194-7) contains supplementary material, which is available to authorized users.

## Background

Primary care [PC] and public health [PH] are viewed as distinct sectors within the health systems of western societies including Canada [1]. Canadian researchers propose that better integration between PC and PH is necessary for a more effective primary health care system to improve health and social outcomes [2]. Other nations have similar aims [3, 4]. In 2012, in the U.S., a report was released calling for better integration of primary PC and PH services arguing that:

the integration of primary care and public health could enhance the capacity of both sectors to carry out their respective missions and link with other stakeholders to catalyze a collaborative, intersectoral movement toward improved population health. [5] p.1.

Most discussion papers that promote greater integration and collaboration between PC and PH maintain that the goals of each sector can be supported by the other. PC can act as a source of critical data and clinical observation that can highlight health issues of potential consequence to PH and its mandate to promote health and prevent disease as well as improve population health. PH, through its assessment of community and population health risks and needs can inform PC practitioners of things to look for in their patients, subsequently assisting in differential diagnoses and improved patient care [2, 6, 7]. Others acknowledge synergies in health promotion that can occur when education within PC settings aimed at behavioural changes to promote health is combined with PH strategies for creating supportive environments that enable healthy life styles and reduce environmental risks [8]. DeVoe and colleagues [9] discuss opportunities for PC and PH collaborations to jointly address the social determinants of health.

In 2013, a special issue of the journal *HealthCare Papers* indicated a continuing interest by influential leaders in Canada for building stronger collaboration between PH and PC sectors [10, 11]. Various influences that impact collaboration between PC and PH sectors presented within the international literature are discussed within a scoping review [12]. However, within this discussion there is limited substantive evidence about the important influences on successful PC and PH collaboration, how these influences relate to each other, and the mechanisms occurring within these relationships.

We report here on one of five studies conducted in a program of research [13] – *Strengthening Primary Health Care through Primary Care and Public Health Collaboration*. The program of research was guided by an ecological framework [14] describing three categories of determinants for inter-organizational collaborations including systemic, organizational, and interactional levels. This paper focuses specifically on factors that influence PC and PH collaboration at the organizational level in the Canadian context. Our results can inform collaboration in countries with similar health care systems. Here, organizational level influences refer to influences at the local or regional level within the context of an organization, large or small. Whereas, systemic level influences are at a national or provincial level such as ministry policies, strategic directions, and funding.

Organizational influencing factors can be thought of as operational attributes, processes or conditions within an organization. Organizational factors affecting collaboration can include, “structure and philosophy, team resources and administrative support, as well as communication and coordination mechanisms” [14] p.138. Our scoping literature review identified five major organizational influences on collaboration between PC and PH [12]. They included: lack of a common agenda; knowledge and resource limitations; leadership, management and accountability issues; geographic proximity of partners; and shared protocols, tools and information sharing. No research papers were found in our review that specifically explored influences on PC and PH collaboration. However, we extracted factors from results and discussions of papers reporting on collaboration. The present study contributes new knowledge by validating our previous review findings and delving deeply into factors explicitly influencing organizational influences on PC and PH collaborations supported by experiences of key informants in PC and PH. It also explores mechanisms that help to explain relationships between influencing factors.

Within Canada, the organizational environment of PC and PH varies depending on the province or territory. For example, Ontario (ON) has public health units while Nova Scotia (NS) and British Columbia (BC) have regional health authorities that provide public health programs and services. There are a variety of PC delivery models in each province [15–17]. In ON, there are 11 models of primary care delivery, such as solo physician practices, community health centres, nurse practitioner-led clinics, and family health networks. In BC, PC is mostly provided by physicians in solo and group family practices with some integrated health networks, and less commonly, health authorities also delivery PC through community health centres, and specialized clinics (e.g., youth health, STI diagnosis and treatment) often by nurse practitioners. NS predominantly has solo and group physician practice models but there are a growing number of interdisciplinary teams, particularly in rural areas. Furthermore, in some instances, PC and PH working spaces and regional reporting accountabilities are shared, while in others, each sector is visibly and operationally its own entity [6, 15]. This diversity creates a rich naturalistic opportunity for further defining the organizational factors influencing PC and PH collaboration. This paper explores: what structures and processes do PC and PH stakeholders perceive influence successful collaboration between PC and PH? Table 1 provides our definitions of PC, PH, and collaboration.

**Table 1:** Definition of Terms

Primary Care: “…the crucial foundation of a health care system, and defines the key features of primary care as being the first point of entry to a health care system, the provider of person-focused care (not disease oriented] over time for all but the most uncommon conditions and the part of the system that integrates or co-ordinates care provided elsewhere or by others.” (Starfield, 1998)	
Public Health: “…an organized activity of society to promote, protect and improve, and when necessary, restore the health of individuals, specified groups, or the entire population. It is a combination of sciences, skills, and values that function through collective societal activities and involve programs, services, and institutions aimed at protecting and improving the health of all people. The term “public health” can describe a concept, a social institution, a set of scientific and professional disciplines and technologies, and a form of practice. It is a way of thinking, a set of disciplines, an institution of society, and a manner of practice. It has increasing number and variety of specializes domains and demands of its practitioners [and] increasing array of skills and expertise” (Public Health Agency of Canada, 2008) p.13.	
*Collaboration*: is defined as: “a recognized relationship among different sectors or groups, which have been formed to take action on an issue in a way that is more effective or sustainable than might be achieved by [any one group or sector] acting alone.” (Public Health Agency of Canada, 2008). p.9	

## Methods

We conducted an interpretive descriptive qualitative study, which is a methodology developed specifically to conduct practice-oriented research in health care [16, 17]. It involves descriptions and interpretations about a phenomenon from the perspectives of those who have lived it, in this case, those who have been involved in PH and PC collaborations. Interpretive description was an appropriate methodology for our purposes because it seeks to develop understandings of practice phenomena (e.g., PH and PC collaboration) that “illuminate their characteristics, patterns, and structure in some theoretically useful manner” [17] (p. 6).

We applied a purposive sampling approach [18] to ensure representation across disciplines, roles, and sectors. Using snowball sampling, we recruited policy makers, managers, and direct service providers in PC and PH, and from a variety of disciplines. Recruitment was done by email with a letter of consent attached; agreement to participate was deemed as consent. Key informants were from three provinces (BC, ON, and NS) and representatives of national organizations. No one refused to participate, however, a few did not respond to invitations. We continued to invite participants and send reminders until we reached comparable levels of participation from each sector and province. Although we did not closely track the number of those approached for participation, we obtained our sample easily. Some participants were only approached once and then not pursued since we had reached our target.

Seventy-four key informants participated including: BC (*n* = 20; 27.0%); ON (*n* = 19; 25.7%); NS (*n* = 21; 27.0%); and national organizations, government, or associations (*n* = 14; 18.9%). Of these 74 participants: 78.4% were female; 43.2% worked in or were responsible for PC, 44.6% worked in or were responsible for PH, 9.5% represented both sectors, and 2.7% worked in neither and were researchers or leaders in professional associations or involved with national policy. Table 2 reports the participant breakdown by sector and region and Table 3 shows their role and discipline. Participants had 5 to 40 years of experience in healthcare; 68 % had over 20 years.

**Table 2:** Provincial Representation of Participants by Sector

Sector	BC n (%)	ON n (%)	NS n (%)	National n (%)	Total n (%)
PC	10 (50.0)	9 (47.4)	10 (47.6)	3 (21.4)	32 (43.2)
PH	10 (50.0)	10 (52.6)	10 (47.6)	3 (21.4)	33 (44.6)
PC and PH	0 (0)	0 (0)	1 (4.8)	6 (42.9)	7 (9.5)
Neither	0 (0)	0 (0)	0 (0)	2 (14.3)	2 (2.7)
TOTAL	20 (100)	19 (100)	21 (100)	14 (100)	74 (100)

**Table 3:** Roles and Disciplines of Participants

Role	Number	Percent
Direct service providers	17	22.9
Senior program managers	14	18.9
Executive officers	11	14.9
Middle Managers	10	13.5
Policy Makers	8	10.9
Other (e.g., health educator, coordinator, consultant, researcher)	14	18.9
Total	74	100
Discipline	Number	Percent
Physicians	14	18.9
Registered nurses (not including public health nurses)	14	18.9
Public health nurses	11	14.9
Business administrators	8	10.8
Nurse practitioners	7	9.5
Other professional disciplines (health promoter, dietitian, social worker, epidemiologist, psychologist, public health dentist, etc.)	20	27.0
Total	74	100

Forty-five to 90 min interviews took place by phone guided by a semi-structured interview guide (see Additional file 1). A core question was “Why do you think some collaborations between PC and PH have worked while others have not?” Prompts were used to explore systemic, organizational and interpersonal factors. Interviews were audio-taped, transcribed, cleaned and anonymized. Researcher co-leads in each province included one PC and one PH expert [LO, DMS, MM, RMM, RV and SW]. Each provincial team collected their province’s data and the ON team conducted interviews at the national level. There were a few instances in which interviewers had past relationships with interviewees due to the relatively small provincial PH communities. Where this occurred, their data were analysed by another team member. All authors have extensive experience in conducting qualitative and publishing qualitative research and research staff had qualitative coding experience.

Coding was supported with NVivo 10 software [19]. Following a careful reading of interview transcripts, two were coded independently by two researchers. First level codes were then categorized into second level codes [20], to create a first draft of a code book. Provincial teams then independently coded another subset of transcripts before meeting with the full team.

We used an interpretive thematic analysis approach [16, 17, 21] drawing on the constant comparative method of grounded theory [22] for inductive coding and analysis because it allows both description and interpretation as analysis proceeds through first level coding to developing categorizations and interpretations of these categories at higher levels of abstraction. A final code book was created through multiple full team meetings where consensus was reached. The code book included three levels of coding including: first level nodes (e.g., information systems for sharing data) which were collapsed into elements (e.g., effective communication strategies) followed by influencing factors (e.g., strategic coordination and communication mechanisms).

Saturation was reached at the level of the elements. Credibility of our analysis was supported by memoing, constant comparison, and continual evaluation with the full team. These techniques helped to expose influencing factors and relationships among them. Matrix queries in NVivo 10 were used to examine potential cross-sectoral and cross-provincial differences. Queries pulled text passages coded for one influence that were located ‘near’ another influence. A manual review of these text passages was conducted to identify potential evidence of relationships and mechanisms among the influencing factors to support our interpretive descriptive approach.

## Results

### Influencing factors on collaboration at the organizational level

Seven organizational influencing factors affected or determined the nature of PC and PH collaboration: 1) Clear Mandates, Vision, & Goals; 2) Strategic Coordination and Communication Mechanisms between Partners; 3) Formal Organizational Leaders as Collaborative Champions; 4) Collaborative Organizational Culture; 5) Optimal Use of Resources; 6) Optimal Use of Human Resources; and 7) Collaborative Approaches to Programs and Services Delivery. Each influencing factor is described by its elements (shown in *italics*); both are summarized in Table 4. Noteworthy relationships among elements are identified as each organizational influencing factor is presented. We also indicate differences by province and sector where evident. Quotes are used to showcase influencing factors indicating the participant’s sector [PC or PH] and province [ON, NS or BC]. ‘Primary healthcare’ is used in quotations when it was used by participants to refer to PC. National level participants or those from provinces outside of BC, ON or NS are identified as ‘national’ along with their sector where applicable [PC, PH, Both, or Neither]. Relationships among influencing factors are addressed in the final section of results.

**Table 4:** Organizational Influencing Factors and Elements in PC and PH Collaboration: Comparison between Study Results and Scoping Review Results [12]

Organizational Level Influencing Factors	Elements of Each Factor from this Study	Comparable Scoping Review Results (*Factors* and related descriptors)
1. Clear Mandates Vision and Goals	• Clear mandate for collaboration • Congruent focus • Formal agreements • Organizational structures that enable collaboration • Role delineation	*Lack of a common agenda* • Lack of a common agenda or vision • Different focus • Lack of joint planning *Leadership, management and accountability issues* • Contractual agreements • Designated staff supporting collaboration • Supportive job descriptions
2. Strategic Coordination and Communication Mechanisms between Partners	• Formalized communication processes • Strategic plan development by partners • Coordinated clinical and administrative services • Exchange of client/health information	*Shared protocols, tools and information sharing* • Shared standardized information systems • Shared protocols re: practice, quality assurance, data collection and dissemination
3. Formal Organizational Leaders as Collaborative Champions	• Ability to move towards a common goal • Leadership buy-in to collaboration • Transformative leadership qualities and skills	*Leadership, management and accountability issues* • Change management • Optimal functioning of healthcare providers • Stable, diverse teams • Management training for supporting collaborative teams
4. Collaborative Organizational Culture	• Valuing the work of the other sector • Organizational readiness for collaboration • Avoiding turf protection	*Lack of a common agenda* • Lack of organizational support • Differences in organizational culture • Devaluing PH activities
5. Optimal Use of Resources	• Investment of resources to initiate and maintain collaboration • Funding mechanisms • Geographic proximity of partners • Time for working on collaboration	*Knowledge and resource limitations* • Financial Resources • Space limitations • Lack of time for collaboration *Geographic proximity of partners* • Co-location to facilitate communication, information exchange, trust
6. Optimal Use of Human resources	• Matched professional skills to needs • Professionals work to optimal scope of practice • Organizational mandates enable working to optimal scope of practice • Flexible, accommodating application of skill sets	*Knowledge and resource limitations* • Human Resources • Needs assessment skills in PH *Leadership, management and accountability* • Optimal functioning of healthcare providers • Stable, diverse teams • Administrative support
7. Collaborative Approaches to Programs and Services Delivery	• Engaged community • Client-centred approach • Inter-professional teams, • Integrated or coordinated programs and services between public health and primary care	*Leadership, management and accountability issues* • Community based committees with diverse membership • Community engagement • Involvement of multiple professionals

#### Influencing factor 1: clear mandates, vision, and goals

Clear Mandates, Vision, and Goals was a key organizational level influencing factor affecting PC and PH collaboration. Its five elements include: a) *clear mandate for supporting collaboration*; b) *congruent focus*; c) *formal agreements*, d) *organizational structures that enable collaboration*, and e) *role delineation.*

Having a clear mandate for supporting collaboration at the organizational level was an important element noted by many participants.

Together you have responsibility to make this place work. [National/Both].

One PC administrator noted that there are mandates in hospitals to collaborate more so than in the community and highlighted the need to strengthen this imbalance:

So organizationally, collaboration became a mandate and became a way of doing things. That hasn’t happened yet in most Health Authorities. And it certainly hasn’t happened at the community level to the extent that there is potential. I think that there’s opportunity for the organization and governance of things to facilitate that at some point. [NS/PC].

Being clear about the mandate of each sector and ensuring that they are well understood by both parties was also important. Misinterpretations about each other’s mandates seemed to be detrimental to collaboration:

If you think population health is [about] acting only at a policy level then you are not going to collaborate with PC, are you? [NS/PH].

This quote reflects a participant’s view that some colleagues have a narrow view of population health that ignores other actions beyond policy interventions, such as early childhood development that can improve the health and well-being of populations.

Similarly, all provinces noted that having a *congruent focus* between sectors was an important element for supporting collaboration. Health promotion, disease prevention and chronic disease management and prevention practices were described as having “a lot of overlap” [National/Both] between sectors. Each sector, however, takes a different approach when addressing the same issue:

The work processes in PC tend to be individual, episodic and, in the case of PH they tend to be quite different in terms of the way that the business process works. There’s a lot more group work, there’s a lot more in the field work and a lot more regulatory [work]. [BC/PH].

Because each sector takes a different approach to health promotion and disease prevention, recognition of this congruent but specialized focus by practitioners from each sector can lead to an understanding of the value of collaborating to cover the full spectrum of practice.

*Formal agreements* were often lacking, but were also seen as a way to support collaborations. For example, one collaboration described a MOU:

So we have what is called an MOU – a memorandum of understanding – of how we work together. So the MOU says that each partner agrees to put 4 h of service in on a weekly basis. And from that memorandum, we have a planning day every year. And so it could be that PH is going to do some immunizations for us.

Participants spoke about the need to develop more formal working relationships for particular issues, such as pandemic planning or influenza outbreaks. The autonomy and independence of PC physicians was perceived to hamper building relationships and subsequent development of formal agreements to work in collaboration with PH. This was related to PC having had a very long history of being in independent practice.

A few participants believed that there were *organizational structures that enabled collaboration* or presented barriers. Most often PC participants spoke of PH’s large bureaucratic unionized organizational structures being a barrier. A PC practitioner noted:

The bureaucracy drives me crazy and the inactivity and inability that happens when you get caught up in meetings and bureaucracy. And you’re unable to act because you are too busy talking about how to reach the sex trade worker and, what are the attributes of a sex trade worker and, rather than getting out there and actually talking, touching, and making connections. [NS/PC].

On the other hand, non-unionized PC environments, such as community health centres, were perceived to be more flexible in how they manage their human resources which enable collaborations.

A PH participant in NS articulated how *role delineation* and communicating any differences in roles between PC and PH was essential for collaboration:

…if we think about any of the roles where PH and PC intersect. Whether it’s community health assessment, immunization, chronic disease, communicable disease, even emergency preparedness, there are certain pieces within each of those that require a PH philosophy and a PC philosophy. And it may be just a matter of sitting down with each program and having a discussion with somebody from PC and PH to say, ‘okay, what do you do under this heading? What can you offer?’ This is where you [PC] would come in. This is where I [PH] would come in. [NS/PH].

Once roles were defined they were documented in formal agreements as noted in an earlier quote. This quote also illustrates the relationship between the elements *role delineation* – being clear about what each sector can contribute - and having a *congruent focus* - applying different approaches to disease prevention and health promotion but each being congruent with the other.

#### Influencing factor 2: strategic coordination and communication mechanisms between partners

Strategic Coordination and Communication Mechanisms between Partners has four elements: a) *formalized communication processes*; b) *strategic plan development by partners*; c) *coordinated clinical and administrative services*; and, d) *exchange of client/health information*.

*Formalized communication processes* were critically important for facilitating collaboration in all provinces and sectors. Effective and ineffective communication processes were reported. Having formalized meetings, case conferences, or other communication processes to ensure regular opportunities to stay connected was a key enabler. Agreeing on a common language was also highly valued in starting collaborations:

Language has played an important role in the division of culture between these two groups and so finding common terminology and words that people can live with and the lens that people are bringing to the application of those words has been very important in doing translation and in finding joint projects. [BC/PH].

The element, s*trategic plan development by partners* was closely linked with *formalized communication processes.* Although this element was not raised often in interviews, participants saw it as being necessary to ensure coordination of programs. The relationship between these two elements is illustrated as follows:

Everybody communicates, collaborates. Do your gap analysis. Say ‘this is what we bring to the table’. Share, and then whoever is best positioned to move an initiative forward does so. And then it is done in cooperation with all the other groups. Then you can pull back and develop your program, and then you come back forward again and say ‘okay, how are we doing’. Rather than the traditional, which is, develop your own program in isolation of everybody else. [NS/PC].

There were a few cases in which PH staff sat on Family Health Teams boards (an ON interprofessional PC team-based model). A PC provider explained:

[PH] are right here when we’re making our most basic decisions of our governance and vision and what we’re looking for, for the following year [ON/PC].

*Coordinated clinical and administrative services* was identified as an important collaboration element distinct from organizational strategic planning, the former being managed at the program delivery level. The following example describes how coordination was needed for service delivery for vaccine programs involving both sectors:

If you’re going to leave it to family docs, you don’t just say, ‘good luck guys go and do immunization.’ You have to actually organize getting them the vaccine. You have to organize them reporting who they vaccinated... [PH/BC].

The above quote supports the element *– exchange of client/health information*. It was reported in all provinces, most often by BC participants. This was often related to sharing patient information (e.g., infant follow up, immunization records) with PH as well as other partners (e.g., home care). A BC PH physician explained that:

There would be more regularized referrals between PC and PH. […] particularly [if you had] more records and electronic medical record sharing between the two sectors [BC/PH].

Not sharing client records was reported more often than sharing. As another physician explained:

We had an automatic relationship with [PC], but often we don’t get reports back from physicians as to what families they’ve immunized and it makes it difficult for our records, etc. [BC/PH].

A barrier for sharing information was the use of different forms of documentation:

There is data collection by PH that we could not piggy back onto. We couldn’t add our notes or assessments [PC/ON].

If PC and PH sectors cannot share data, it is difficult to collaborate effectively.

#### Influencing factor 3: formal organizational leaders as collaborative champions

An important influencing factor supporting collaborations is having Formal Organizational Leaders as Collaborative Champions. This includes the elements: a) *ability to move towards a common goal*; b) *leadership buy-in to collaboration*; and, c) *transformative leadership qualities and skills*. This factor was less commonly raised by participants compared to other factors; nonetheless it was identified by some participants in each province and sector.

The element - *ability to move towards a common goal* describes attributes needed by organizational leaders to have the power to move collaborations forward. One such attribute is the importance of having a vision:

So, if the leader doesn’t have a vision of what it’s going to look like then they’re not going to lead the way. [PH/ON].

Middle and senior level managers were identified as leaders with a role in enabling collaborations:

And it’s up to the managers, I believe. That is a key role of directors, but especially the managers, to create the environments to allow that to happen. [NS/PC].

*Leadership buy-in to the collaboration* was viewed as another significant element in successful collaborations. Having leaders at a senior level who “really believe in it” was essential for collaborations to work, whereas, a lack of leadership buy-in was a barrier. A BC PC participant described wanting to share immunizations records for his older adult patients with PH nurses, however, the regional health authority was unsupportive:

[the health authority did not] see [delivering immunizations to older adults] as their role. They don’t see that there’s any importance to that. And so it really… hampers community-based provision of appropriate care to people at risk. [BC/PC].

This quote illustrates a relationship between factors. The example illustrates how PC leadership buy-in around collaboration for immunization data exchange was obstructed by PH’s organizational mandate that excluded tracking older adult immunizations. Some participants also noted that it can be challenging when leadership changes, which can negatively influence the commitment towards collaboration.

Although not explicitly named as such, some participants spoke about *transformative leadership qualities and skills* that were needed to support collaborations:

…a more democratic, open, sort of leading from the heart, not just the head type of approach. So the ability to put yourself in each other’s worlds and understanding where people are coming from. […] And recognizing that everybody has a part to play, and that one role isn’t more important than the other. But all together, we can make such a difference, a positive impact on the outcomes for clients, for communities, for populations. [NS/PH].

Transformative leaders consider the value brought by each player within the collaboration to ensure optimum use of human resources to support collaborations, which is another factor to be discussed later.

#### Influencing factor 4: collaborative organizational culture

Having a Collaborative Organizational Culture is an essential influencing factor for supporting collaboration at the practice level. It consists of three elements: a) *valuing the work of the other sector*; b) *organizational readiness for collaboration*; and c) *avoiding turf protection.*

*Valuing the work of the other sector w*as a strong influence on collaboration, identified as being essential by both sectors and all provinces. A condition for valuing the other sector was having an understanding of it. As one participant explained:

There is a lack of respect sometimes for primary healthcare providers. If people understood what [PC has] to deal with day-in and day-out and the volume of work, there would be more understanding. [NS/PC].

PH also felt misunderstood and wanted to increase their credibility with their PC partners. One area that was misunderstood was related to:

…the importance of PH and prevention within the context of chronic disease and its management. [ON/PH].

PH was also concerned about being perceived as having a more passive and undervalued consultant role rather than a more active role:

I think the whole world wants to see PH actually do something. PH [has] to show themselves to be credible. And they’re not credible by handing out pamphlets. I think that all PC people are looking to have a partnership where PH doesn’t see themselves as a consultant but sees themselves as a worker […] prepared to get their hands dirty. [ON/PH].

*Organizational readiness for collaboration* was often identified, in all provinces and both sectors, as a positive influence on collaboration. Participants reported several examples of existing collaborative working groups. Readiness to collaborate was associated with having common goals and values:

… the goals and the principles and values as well are important to have, so that people … are thinking of things in a similar way. [ON/PC].

A lack of readiness was attributed to rigidity of practices in PC and PH. This generally related to PC physicians who were too busy for collaboration, and PH being too structured and unprepared to meet PC’s needs.

We would like a PH nurse to come out 4 h a week to do a breast feeding clinic. And it actually got turned down because they thought if they did it for us, they might have to do it for other clinics too. [NS/PC].

*Avoiding turf protection* was raised by several participants across sectors and provinces. But most often was expressed as PH protecting their turf.

When some of our ‘primary healthcare’ people get into prevention… PH is saying, ‘That is ours.’ [NS/PC].

PH’s turf protection was considered by some to be a response to their fear of losing resources as captured by this sentiment:

…We [PC] want to work with you. And they say: ‘Just a minute now. I’m a little worried when you say that because typically what that means to me in the past is to come along and take away. Take away our business, take away our resources.’ [Nat/PC].

#### Influencing factor 5: optimal use of resources

Collaboration is very difficult without adequate fiscal, material and space resources. Given the difficulty experienced by both sectors in obtaining resources for collaboration, any resources that are available must be used optimally. Optimal Use of Resources consists of four elements: a) *funding mechanisms;* b) *investment of resources to initiate and maintain collaboration*; c) *geographic proximity of partners*; and, d) *time for working on collaboration*.

*Funding mechanisms* that support collaboration was a commonly identified concern for PC as well as PH.

Unfortunately, in an effort to perhaps reconcile and protect [PH’s] scarce resources, we are finding a pretty strong line about not only what they will do or not do but what they will even be involved in planning. [NS/PC].

PC practitioners face their own funding constraints which dictate what activities they take on creating challenges for PH:

So [PH is] not quite sure about how to connect up with the [PC] system where people don’t work that way. I mean, of course, [PC does not] pay somebody, they don’t get paid (to collaborate) and so [PH] feels awkward to try to get to [PC] to loosen up time when they’ve got bills to pay and staff to pay and so on. [BC/PC].

Funding for collaboration is not ensured in either sector.

A related element, *investment of resources to initiate and maintain collaboration* is required by both sectors. This is a particular challenge for non-salaried PC payment structures, which is explained as follows:

…if I wanted to bring a PH nurse out to have a home in our clinic 4 h a week, logistically there [are] overhead costs associated. We have computerized patient records. So they would need a computer. They would need supplies and equipment. The receptionist would be checking in patients so there is additional workload. They would need a phone. And that is because they (PC) are private businesses right now. It’s fee-for-service. So they have to pay for everything that happens in that clinic. [NS/PC].

For PH, resources investments were most often tied to time. As expressed by a health promoter:

I think an acknowledgment from management to senior management to funders of the amount of time and dedication that it takes to develop, sustain and maintain collaborations. That’s critical…. to develop and sustain. (ON/PH).

*Geographic proximity of partners* was generally described as an enabler for collaborations:

There’s many other small examples of collaboration. One of them is the fact that ‘primary healthcare’ and PH administratively are side by side in the same corridor which allows for greater collaboration. [NS/PC].

Physical proximity was viewed as a support to building relationships through increased face time that also made for easier referrals. Although reported less often, geographic distance was viewed as a barrier. Some participants suggested that people need to be in a common network if not in the same space. Not sharing space led to inefficiencies:

Unfortunately, the nurses that were there were kind of bopping back and forth between the two places carrying their records with them. And it just became very difficult for them. Ultimately, we would like to have a one site vision where we would all be in one site under the same roof. [Nat/PC].

*Time for working on collaboration* was presented as a barrier. Despite being a less commonly identified element, it was noted in each province and sector. Time was needed to get to know and understand the other sector as well as communicate with collaboration partners. Time became a bigger challenge when working with PC teams in collaborations. As noted by a PC business administrator:

There’s a cost and energy to that communication. […] thinking that you were, for example, in a community health center and you had a team of eight people. The number of times you have to communicate to be clear is totally different than if you only have two people [BC/PC].

Giving time to collaborations has monetary tradeoffs that need to be acknowledged. For physicians,

their income depends on moving clients through their fee-for-service system. We’ve had more success breaking down that barrier, if we can provide them with auxiliary staff to support the project. [BC/PH].

This relates to the need for dedicated human resources to support collaborations - the next factor.

#### Influencing factor 6: optimal use of human resources

To enable collaboration in systems with scarce resources, it is essential that human resources be used effectively to optimal scope to support the goals and work of the collaboration. Optimal use of Human Resources has four elements: a) *matching professional skills to needs*; b) *professionals working to optimal scope of practice*; c) *organizational mandates that enable working to optimal scope of practice*; and d) *flexible, accommodating application of skill sets*.

Participants expressed the need to ensure that there are *matched professional skills to needs,* thereby, ensuring the staff have the skills required to address the needs that are the focus of the collaboration. One participant presented an example of how PC and PH collaborations use a range of professional skill sets to address population health needs:

They’re trying to get some synergies out of the program. If the PH dieticians end up with some people participating in their programs that actually need a little bit more counselling, they can refer them onto the dieticians in Family Health Teams. PH dieticians are a little bit more adept at understanding the Canadian community health data statistics that come out. And so they can interpret those and work together to try to address the needs in the community. [NAT/Neither].

The element*, professionals working to optimal scope of practice*, requires an understanding of each other’s scope to maximize the use of human resources:

We really want to see PC services delivered according to many different models; some based on general needs, others on population health needs. So, that requires inter-professional collaboration. And the roles of registered nurses in PC and PH really being well understood and nurses being able to work to their full scope of practice. [NAT/BC].

To ensure *optimum scope of practice*, participants acknowledged the need to *match professional skills to needs* so that the right people were in the right place at the right time.

Another element, o*rganizational mandates enable working to optimal scope of practice,* means allowing professionals to work using the skills for which they are specifically trained:

… one of the things that I think is so positive about ‘primary healthcare’ models is that it’s taking that pressure off one or two providers to do everything for everybody. But the benefit of that larger team to share the responsibility and the patients. I think that is a tremendous help to seeing it from again a healthier, more balanced perspective and then you can start thinking about the collaboration. [BC/PH].

*Flexible, accommodating application of skill sets* implies a willingness to do what’s necessary to make the collaboration work:

So, sometimes you just got to pitch in and do the dirty work together. And they did it. Those nurses were incredible that did that. See, that’s not your [job]. No one would sign up for that. It was time limited and they thought [it was] a way of building the partnership. [ON/PH].

*Organizational mandates enable working to optimal scope of practice*, and, *flexible, accommodating application of skill sets*, were identified as related elements that influenced each other. Both elements were further linked to *professionals working to optimal scope of practice* as described above. The relationship among these elements was summarized as follows:

So organizational mandates do get in the way of collaborative work. We need to know what our subsequent roles are, absolutely, and what our boundaries and scope of practice is. But within that, there needs to be flexibility to work with the community. So that dietician wasn’t going to be doing something outside of her scope of practice [for example] to go to this wellness day. And the flexibility to be able to enable that. It was determined that it was a good idea to do an 18 month wellness [assessment]… for the PH nurse to be able to go work with that family practice and not say, ‘No, that is the family practice’s thing. They’ve got a nurse practitioner. She can do it.’ [NS/PH].

Organizational mandates are identified as a barrier to collaboration in the quotation above. On the other hand, establishing organizational mandates that encourage collaboration could ensure that partners work together with positive outcomes. In summary, organizational policies need to allow for flexibility in practice balanced with providers working to optimal scope of practice, thereby allowing collaborative work to flourish.

#### Influencing factor 7: collaborative approaches to programs and services delivery

The final influence on PC and PH collaboration is ensuring that the approaches to programs and service delivery facilitate collaboration. Collaborative Approaches to Programs and Service Delivery consists of four elements: a) *engaged community*; b) *client-centred approach*; c) *inter-professional teams*; and*,* d) *integrated or coordinated programs and services between PH and PC.*

The element *engaged community* refers to working with communities directly in program planning, development and delivery. This concept was raised more by PH than PC and by participants in BC compared to other provinces. This element was identified most often in relation to working with marginalized populations and often referred to using community development approaches in collaborative work. Community development activities that require engagement by community members was identified as a potential strategy for PC and PH collaborations. A NS PHN explained:

A lot of PH staff have been trained in community development. They could be that [dedicated resource] person who makes the links between all the pieces of the system [NS/PHN].

A PC physician spoke about leveraging partnerships with other community organizations, including PH, to apply for funds for collaboration. In addition, *engaging community* made the most sense where PC and PH both served a specific geographic community. As noted by one national level participant:

...you have to be able to bring it up to a community area level […] So, you need to bring all your clients together and then look at what the community needs. [Nat/Both].

A *client-centred approach* is an element identified and applied by community health centres, which provide interprofessional care to marginalized populations. Providers in these settings tend to focus on the specific needs and assets of individuals:

It is the whole client focus that is so central to the whole community health centre way of thinking. [PC/ON].

This approach was key to PC and PH collaboration because the motivation to collaborate then focuses on client-centred health goals that are understood and common in both sectors.

*Inter-professional teams* is another element for effective collaboration acknowledged in all provinces and both sectors, although it was not commonly reported. PC and PH participants described inter-professional teams in their organizational contexts that could support collaborations. In BC, participants commented on the historical lack of resources and supports for team formation and new mandates for them to work inter-professionally. A few commented that PH staff such as health promoters, PH nurses, and epidemiologists could contribute to PC through potential secondments. Some felt that it would be easier to collaborate with teams rather than independent practitioners:

I would assume that in Family Health Teams, particularly where there are more disciplines that are represented, that the coordination and collaboration with PH is probably easier than in those family health teams that only have physicians or nurses [PH/ON].

The final element for this category of influence on collaboration is the appeal of *integrated or coordinated programs and services between PH and PC.* Although this was desired, most participants reported that in reality the two sectors work in silos rather than in an integrated fashion:

If you were meeting with [PH] and saying: ‘We have this set of population, these people. Who could do what to serve those people best?’ But I think we are still very much in our own little silos [NS/PC].

Some participants spoke about the need for incentives to increase the development of integrated or coordinated programs and services:

…you have to incentivize getting group practices together. And I think one of the ways you can incentivize a group practice is by providing to a group practice PH services. But that will require an expansion of PH services to be able to meet a growing demand. [BC/PH].

In summary, engaging clients, ensuring a client-centred approach, using inter-professional teams and building integrated programs can help ensure that a collaborative culture exists to support successful collaborations.

### Relationships among influencing factors

**Fig. 1:**
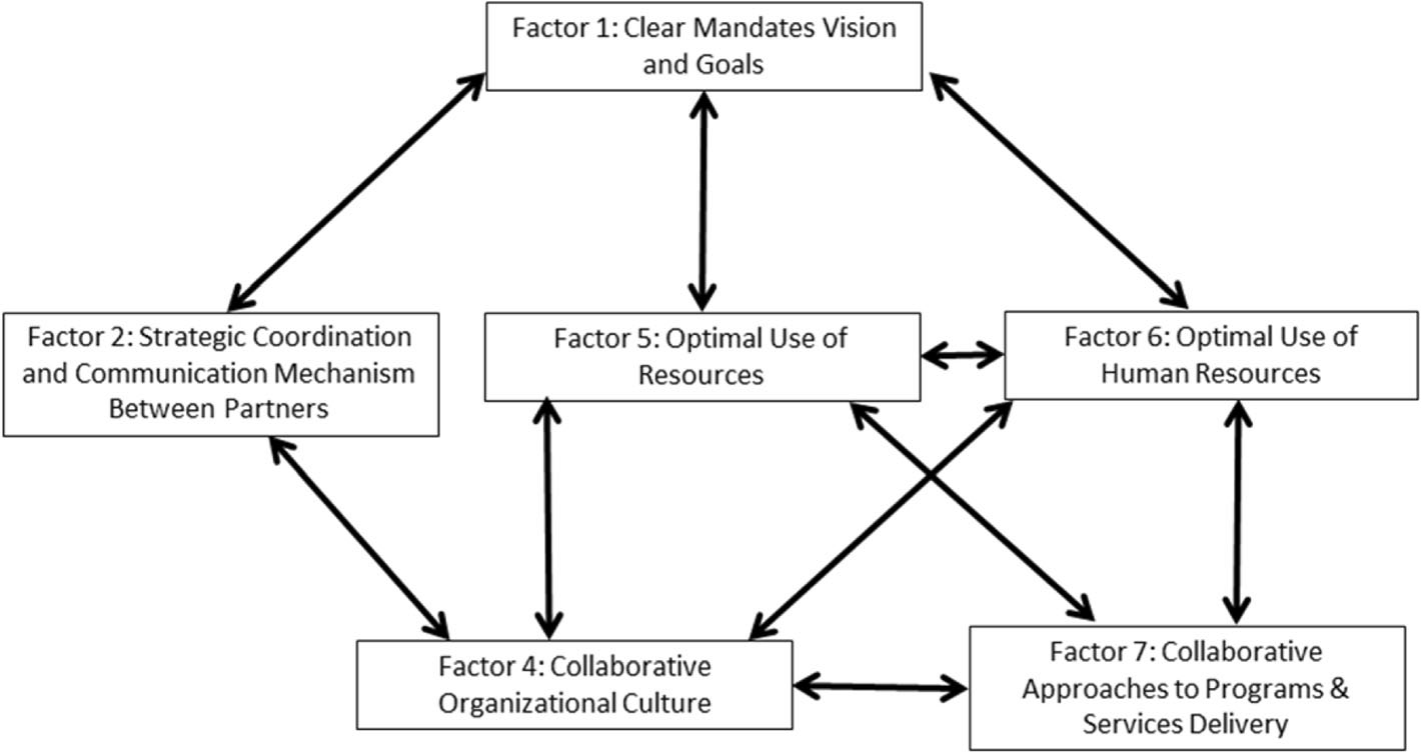
Commonly reported relationships among influencing factors for primary care and public health collaboration

All seven organizational influencing factors were found to interact with each other although some were noted less often in our data. For the sake of brevity, we only highlight interrelationships among influencing factors where they were most apparent in our results (Fig. 1) and pose possible mechanisms that explain these relationships. Clear Mandates, Vision, and Goals (influencing factor 1) interrelated most readily with all other factors. The relationship between this factor and Strategic Coordination and Communication Mechanisms (influencing factor 2) is described as follows:

...if you start with the leadership and the vision then you need to have your processes in place. Having a team that’s knowledgeable enough to know what needs to be integrated. What would promote collaboration, like the agreement that we talked about, or having the same phones, the same computer system for their whole information technology that promotes collaboration. [Nat/Both].

The above quote illustrates that having a Clear Mandate, Vision and Goals for a collaboration is required to support the development of Strategic Coordination and Communication Mechanisms to support the collaboration to move forward thus identifying the temporal nature of the relationship between these factors. The following example further illustrates the nature of these interrelationships and how the first influencing factor drives the second.

[Having] a common vision, identified common goals. If there were a collective of primary caregivers around the local [PH] unit, [and] there was an agreement that low birth weight rate in the city that you live in would go from six to five or seven to five or whatever, with common planning, that would work. [ON/PH].

This quote illustrates how having a congruent focus*,* an element of the influencing factor, Clear Mandate, Vision and Goal, drives joint decision-making to set measureable goals. In turn, this informs joint strategic planning and coordination processes, an element of influencing factor 2, Strategic Coordination and Communication Mechanisms between Partners). The reverse relationship also exists. For example, lacking Strategic Coordination and Communication Mechanisms can negatively influence the development of clear goals for a collaboration. A participant explained how strong communication mechanisms are needed to develop common goals to begin a collaboration:

People who work in those two different settings are just oriented to those different approaches. So, to bring them together to solve a mutual concern…and I think that’s one of the other issues is that PH and PC, from my experience, have rarely been brought to the same table to address a common issue. [ON/Both].

Clear Mandates, Vision, and Goals (influencing factor 1) was also tied to Optimum Use of Resources (influencing factor 5) and Optimal Use of Human Resources (influencing factor 6). A business administrator explained:

PH has all-embracing vision statements. So I think as both groups started to think a little more about what really is our role and where can we make the greatest impact, [there was] some kind of refinement of those visions and concepts. I think as both realized that to work together that you can no longer be doing the same thing. So I think part of it has been driven by resources, not just money, but human resources. And having to look at just to practice differently, away from the family doctor, everything - to family practice nurses and practitioners. And people were more open to what could happen to work better together. [NS/PH].

The goals and vision that a collaborative initially identifies often will require revision based on available human resources and *flexible, accommodating application of skills* (element of influencing factor 6- Optimal Use of Human resources). This relationship also worked in reverse:

They’ve never had these resources available to them and they’ve not had to think about changing the way they do business to incorporate other team members. [BC/PC].

The new influx of resources forced them to rethink their goals and how to work together.

Collaborative Approaches to Programs and Service Delivery (influencing factor 7) is related to Optimal Use of Human Resources (influencing factor 6). This relationship was aptly described by a national leader in PC and PH:

We really want to see PC services delivered according to many different models; some based on general needs, others on population health needs. So, that requires inter-professional collaboration and the roles of registered nurses in PC and PH really being well understood and nurses being able to work to their full scope of practice (Nat/Both).

This quote highlights the benefit of working in *interprofessional teams* (element of influencing factor 7) that is leveraged by the use of collaborative approaches, such as *organizational mandates that enable providers to work to optimum scope of practice*, (element of influencing factor 6). By promoting an understanding each other’s roles, this element also links to *valuing the work of the other sector*, (element of influencing factor 4- Collaborative Organizational Culture). Increasing the understanding of each other’s roles and functions can correct negative misconceptions and fill knowledge gaps thereby increasing appreciation of the value added by each sector.

A BC public health administrator provided an example of how the relationship between Optimum Use of Resources (influencing factor 5), Optimal use of Human Resources (influencing factor 6), and Collaborative Approaches to Programs and Service Delivery (influencing factor 4) affect each together.

Younger physicians and practitioners in general coming out are getting more used to work in group practices. […] You have to incentivize getting group practices together. And I think one of the ways you can incentivize a group practice is by providing to a group practice PH services. But that will require an expansion of PH services to be able to meet a growing demand then, and it would require some level of funding. (BC/PH).

PC could see how linking PH human capital supported through additional funding could contribute to interprofessional and inter-sectoral practice models.

A relationship between *understanding and valuing the work of the other sector* (element of influencing factor 4 - Collaborative Organizational Culture) and *geographic proximity* (element of influencing factor 5 - Optimal Use of Resources) is illustrated as follows:

There’s a complete difference in socialization that leads to a major barrier in understanding between physicians and other staff. And that is probably the most huge barrier. And then, of course, just the fact that they’re not in the same location [BC/PH].

This quote illustrates how the unique socialization of physicians, which is exacerbated by physical separation from other sectors, isolates disciplines contributing to a poor understanding of one another.

Finally, *collaborative organizational culture* (influencing factor 4) was found to be influenced either positively or negatively by the presence or absence of *strategic coordination and communication mechanisms between partners* (influencing factor 2). For example, a physician shared a scenario in which lack of communication and a siloed culture reinforced strong divisions between sectors and programs:

You find new stuff and you develop a program around it. Unknown to you, you do that [in PH]. But the same program is also being built or has been built in [PC]. If you are not discussing and communicating, you don’t know that each other has this going on. Once you’ve gotten into it and you start developing it, you develop a certain ownership of it in terms of protection, and the empire is built. [NS/PC].

The quote also points to turf protection that can result in the absence of a *collaborative organizational culture* and *strategic coordination and communication mechanisms*.

## Discussion

Participants in this study identified seven key organizational influencing factors that contribute to the success of PC and PH collaboration. While each influence was distinct, many interactions among factors are indicative of the complex and interconnected nature of PH and PC collaboration. This study contributes a rich understanding of these interactions and the potential mechanisms that are at play. The study also provides specific examples of how these influencing factors work in PC and PH collaborations, which can be transferred to others planning or working to sustain such collaborations. Finally this study validates results from our earlier scoping literature review on PH and PC collaboration [12]. Seven influencing factors identified in this current study aligned with five factors found in our scoping literature review.

As seen in Table 4, the results from both studies emphasized different factors with respect to the hierarchy of influences on interorganizational collaboration, (overarching factors vs. subordinate elements or themes). The influence of organizational culture, for example, although receiving mention in our scoping review, rose above other constructs to become a separate identifiable influencing factor from the perspective of our study participants. Indeed, an element of the influence, *avoiding turf protection*, speaks to the dilemma faced by both PC and PH organizations in wanting to collaborate but being challenged to do so when both are reliant on scare resources designated for community health compared to funding available to institutional health care [10, 15]. Turf protection can also arise from the perceived power that one organization has over the ‘other’ for resources that are tied to their mandated roles pointing to it. Walker and colleagues [23] explored risk, trust and control in PC partnerships in Australia. These partnerships aimed to support integration between PC and other community-based organizations. They argue that when organizations work collaboratively they give up some control over their actions and expose themselves to the consequences of other organizations’ activities. This can result in potential harms or risks that must be managed. Partners are driven to protect what they have, and may see ‘overlaps’ in their work as counter to their defense for continued funding [24], rather than as opportunities for collaboration.

Other research published since the scoping literature review validates and expands on our findings. Organizational influences on collaboration identified in our research and supported by the work of others include: the importance of sharing of health data and compatible information systems [25–28], and developing mechanisms and structures for coordination and inter-organizational communication [29, 30]. Data sharing along with effective communication and coordination structures (influencing factor 2) enabled opportunities for leveraging the distinct strengths of each sector. For example, in an immunization campaign carried out in Colorado [29], PH took responsibility for a patient recall/notification program, a population health measure, while PC received patients for administering vaccines, offering individual health care. Despite positive reports in our study as well as other research related to successful exchange of client information, much more work is needed to close the data sharing loop.

Sharing of resources to deliver programs (influencing factor 7) may be an incentive for collaboration [29, 31] although designated or realignment of resources (i.e., funding) for collaboration (influencing factor 5) are also needed [12, 15, 27–29], as well as human resources to support collaborations (influencing factor 6) [5]. With respect to optimal use of resources (influencing factor 5), geographic proximity or use of shared space was often highlighted in the literature as important resulting in synergies for achieving both PC and PH service objectives [11, 12, 15]. Clinical services continue to be offered through some PH organizations in Canada including clinical services for sexually transmitted infections and other communicable diseases, immunization clinics, as well as maternal child health and travel health services [15, 32]. Quite often these services are provided to high risk populations or in areas where PC service gaps are evident [6]. Although these are not necessarily collaborations with designated PC organizations, they do demonstrate the benefit of clinical services operating concurrently with population-based PH programs as part of a population health strategy. Shared space between PH and PC has enabled opportunities for sharing administrative and other costs while more appropriately assigning and matching human resources to need, hence augmenting each other’s talents while enabling practitioners (e.g., PHNs, nurse practitioners, physicians) to operate to their full scope of practice [33]. This illustrates the significant interrelationship between the influences – Optimal Use of Human Resources (influencing factor 6) and Optimum Use of Resources (influencing factor 5) and their effects on successful collaboration.

The current study supports the view that having a Clear Mandate, Vision and Goals for collaboration (influencing factor 1) is a key factor in enabling collaborations between PC and PH. How we interpreted this requires discussion, however, knowing that organizational mandates are influenced to a degree by provincial standards and Ministry directives that do not necessarily outline a mechanism for collaboration [34]. Another paper from our group (Wong et al., submitted) explores the interactions among systems and organizational level influences. In a complementary study under the same program of research, there were differences of opinion among practitioners and government representatives on the importance of mandates [28]. How organizational mandates are operationalized locally was not considered, and may explain some of the disagreement. What did reach consensus in support of collaboration was having a shared vision, as well as a means of interpreting mandates that allowed for ‘blurring of the lines’ between the sectors.

As another paper suggests, changes to legislated mandates at the provincial level can take considerable time (i.e., 10 to 20 years) [24], and are not likely responsive to more immediate needs and/or opportunities for collaboration on issues of common interest to both PC and PH. Instead, collaborations reported to be successful were often empowered through the ingenuity and constructive planning witnessed at the local level, targeting specific health-related activities, and bringing together community stakeholders beyond PC and PH.

In the cases presented in the literature, PC and PH roles are articulated and their specific skill sets and resources utilized toward a common objective. Examples include: immunization [29, 31], obesity campaigns [8], infectious disease and syndromic surveillance [25, 27], sexually transmitted infection management [10, 35] and diabetes prevention [36]. In these examples, a culture of collaboration (influencing factor 4) is encouraged through an awareness of each other and what each can offer [5, 12, 35]. This also ties to formal organizational leaders as collaborative champions (influencing factor 3) at the local level that encourages participation in co-planning initiatives. For example, in New York City, the PH authority worked with the Institute of Family Health representing 26 non-profit health centres, to develop a list of health priorities for targeted communities; priorities were then addressed through campaigns (i.e., ‘Take Care New York’) and patient interventions in a collaboration between the PH authority and PC providers [25].

Two elements under Collaborative Approaches to Programs and Services Delivery (influencing factor 7), continue to be recognized as contributors to collaboration between PC and PH – community engagement, and inter-professional teams or inter-professional collaboration [5, 6, 37]. Our program of research began with the WHO’s definition of primary health care, with recognition that a true model of primary health care encompasses more than just designated health care providers, but other sectors that can and do influence the health of communities [38]. Opportunities for this are more likely to exist within the local context where multiple stakeholders are apt to witness similar concerns, geographic distances are less of a barrier, and community members demand better services through coordinated effort. Broader, more inclusive participatory approaches can be enablers for collaboration between PC and PH in response to a collective community agenda [5]. That said, aligning PH staff to legislated programs rather than geographically designated neighbourhoods may deter PH from participating in local community initiatives [28], a concern also raised in this study.

Collaborations in most reported instances in the literature were very purposeful and project-based; agreements concerning resource needs and strategies on how to proceed were set out by the partners involved in implementing the plan within their own communities. However, for a sustained commitment toward collaboration, formalized relationships could have advantages for encouraging supportive funding, structures and processes. As Walker and colleagues [23] point out, formal governance structures, contracts, and policies that enable tracking and rewarding performance in collaborations is a way that power among organizations can be exercised to regulate the partnership. Further, as stated in the IOM report [5]:

At a minimum, each partner should be committed to a shared goal of improved population health and be willing and able to contribute to achieving that goal. The contribution may range from ideas and planning assistance, to financial or human resources, to goods or a physical space, but ideally will include a shared vision for an ongoing and sustainable relationship and a continual dialogue that goes beyond a single project. (p. 29).

As noted in our results, *optimal use of resources* (influencing factor 5) and *optimal use of human resources* (influencing factor 6) were tied to *collaborative approaches to programs and service delivery* (influencing factor 7). This may be most true for clinical services or programs offered by both PC and PH, for example, immunization clinics [29, 31]; this can also apply to chronic disease prevention programs [36]. Based on results from our study and others, opportunities for collaborations are enhanced when resources and staffing are assigned to support the collaboration. Palinkas et al. [39] explored barriers and enablers in the provision and sustainability of a collaborative care model in PC with mental health organizations for underserved populations. Their results indicated that added workload for clinical staff, delays in information sharing, and lack of resources to sustain the program created collaboration barriers. As in our study, PC physicians whose practices follow a fee-for-service service delivery model [15] are often hampered from participating in collaborations whereas PC settings consisting of interdisciplinary teams are more apt to optimize the scope of practice of the different disciplines through selective assignment of staff to collaborative service initiatives [6].

Notably in the three different provinces participating in the study, different models of community-based PC have evolved, such as Community Health Centres, with different funding schemes that can enable greater community engagement and consequential collaborations with PH and other community partners [6]. However, it is important to note that despite these differences, the influencing factors were seen in all provinces with one exception. Engaged community was raised more often in BC than other provinces which may be related to the particular BC key informants who we included. For example, one participant held community development in his portfolio. Another possibility is that community development and coalition building are noted in BC evidence reviews which are used to guide PH practice.

### Strengths and limitations

We conducted a descriptive interpretative qualitative study with multiple sites; the biases of individual coders may have influenced results, although all coding schemes were ratified through discussions among research team members. We used a snowball sampling technique which can lead to sampling of participants with similar views and those who agreed to participate may have had a natural bias towards the topic. However, although the collaboration influences identified in this study were supported by many respondents, their experiences with these influencing factors were mixed – some positive and some negative - indicating that our sample was represented by people with varied experiences and views. We did not obtain feedback from our participants. There are conflicting opinions in the literature about whether this is advisable or appropriate [40, 41]. However, the results validated those reported in our scoping review [12] and have been validated by the findings of other studies. The different organizational structures and programing offered by PC and PH in the different provinces, although rich in context, added complexity to the interpretation.

## Conclusions

Given that all influencing factors on collaboration identified through our research were related to other factors affecting collaboration between PC and PH, practitioners and managers in organizations need to take all influences at different levels into consideration when planning for or implementing a collaboration. No category of influence should be ignored, although some influencing factors may have more importance at various points in the evolution of a collaboration from the development phase to the implementation and evaluation of the collaboration. For example, the development of clear mandates, vision and goals for the collaboration, anticipated in response to shared interests, would likely be more apparent at the development phase, but needs to be continually communicated and refined over the lifetime of a collaboration. In addition, understanding relationships among influences on collaboration, which are often two-way relationships, is vital for managers and providers working in collaborations. For example, having formal leadership for collaboration will be influenced by and will have an influence on the presence of a collaborative organizational culture.

The seven influencing factors on PC and PH collaboration as identified in this study align with the results of our scoping literature review [12] as well as that of other current research that validate these factors. They also provide more depth in understanding of these various influences, with examples that are specific within the context of the Canadian experience. With sensitivities toward these influences, successful collaborations are more likely, along with the potential for a sustained relationship between PC and PH organizations.

In two companion papers, we consider the influences of systems level [42] as well as interpersonal level factors on collaboration [43]. In two forthcoming companion papers, we explore the very nature of successful collaboration including the structures and processes and characteristics of collaboration, and a final ecological model for successful collaboration (see toolkit2collaborate.ca) highlighting the interrelationships across all levels (systemic, organizational, inter and intra-personal levels) situated within the context of the nature of the collaboration. As suggested in our discussion, systemic change is acknowledged to take time relative to the dynamic of local level processes, opportunities and evolutions within communities requiring a more responsive network of service organizations. Supportive organizational influencing factors on collaborations operating between and within local PC and PH providers can and have, in fact, jump-started collaborations locally across Canada and elsewhere. Key influences have been acknowledged in this more current research with greater emphasis placed on supportive organizational cultures that engage community stakeholders and enable collaborative planning of services and programs directed by a common vision, and with PC and PH practitioners empowered to work within their full scope of practice.

## Additional file

Additional file 1: Semi-structured Interview Guide. (DOCX 18 kb)

## Abbreviations

BC: British Columbia
NS: Nova scotia
ON: Ontario
PC: Primary care
PH: Public Health
