# Supplementary material for: Organizational factors influencing successful primary care and public health collaboration
Source: BMC Health Serv Res. 2018 Jun 7;18:420. doi: 10.1186/s12913-018-3194-7 (PMC5992823; doi:10.1186/s12913-018-3194-7)
Supplement: Supplementary file 1 — Semi-structured Interview Guide. (DOCX 18 kb) [file 12913_2018_3194_MOESM1_ESM.docx]

Addition File 1

**Semi-structured Interview Guide**

This file contains the contents of the interviewee’s guide for the semi-structured interview.

**ID # __________________**

**Strengthening Primary Health Care**

**through Primary Care and Public Health Collaboration**

**INTERVIEW GUIDE**

*The purpose of this interview is to hear your experiences as well as your thoughts and opinions about strengthening primary health care through collaborations between primary care and public health.*

Review the consent form and answer any questions the participant may have.

*First, I would like to get some basic information about you.*

1. **At what jurisdictional level do you currently work?**

__ National

__ Provincial

__ Territorial

__ Regional (Regional Health Authority or LHIN)

__ Local (Municipal/ District Health Authority)

__ Other (please specify) _________________

1. **Which best describes your discipline? (Check one)**

__ Business administrator/ Business manager

__ Community developer

__ Data analyst

__ Dentist

__ Dental hygienist

__ Dental assistant

__ Dietitian

__ Epidemiologist

__ Environmental health coordinator

__ Family home visitor

__ Health promoter

__ Health educator

__ Nutritionist

__ Dietician

__ Speech/language pathologist

__ Pharmacist

__ Psychologist

__ Physician

__ Program evaluator

__ Public health nurse

__ Public health inspector

__ Public health dentist

__ Librarian

__ Nurse practitioner

__ Registered nurse

__ Registered practical nurse

__ Toxicologist infection control practitioner

__ Other (please specify) ____________________________________

1. **Which best describes your main job function? (Check one)**

__ Executive officer

__ Medical Officer of Health/Associate Medical Officer of Health

__ Senior program management

__ Middle management

__ Direct service provision

__ Research /Program evaluation

__ Data analysis

__ Policy development

__ Education

__ Coordination

__ Other (please specify) _____________________________________

**4. Sex** ___ Male ___ Female

1. **How many years have you been in the health care profession**? ________________
2. **How many years have you been in your current position**? __________________
3. **Very briefly describe how you have personally been involved in primary care and public health collaborations.** Consider your current and past experiences.

**Building and Maintaining Collaborations**

*We are interested in your ideas about building and maintaining collaborations between primary care and public health. When we refer to primary care, we are taking into account all models of primary care that provide first contact entry to the health care system such as family health teams, community health centres, fee for service models, family practice networks, etc.. When we ask about public health, we are referring to those who offer population/public health services. When we talk about collaborations we mean any kind of collaboration.*

*Below are some definitions that may help to help clarify the terms.*

*Primary care* has been defined as the:

“…the crucial foundation of a health care system, and defines the key features of primary care as being the first point of entry to a health care system, the provider of person-focused care (not disease oriented) over time for all but the most uncommon conditions and the part of the system that integrates or co-ordinates care provided elsewhere or by others.”

Reference: Starfield B. Primary care: balancing health needs, services and technology. New York: Oxford University Press, 1998.

*Public health* is defined as:

…an organized activity of society to promote, protect and improve, and when necessary, restore the health of individuals, specified groups, or the entire population. It is a combination of sciences, skills, and values that function through collective societal activities and involve programs, services, and institutions aimed at protecting and improving the health of all people. The term “public health” can describe a concept, a social institution, a set of scientific and professional disciplines and technologies, and a form of practice. It is a way of thinking, a set of disciplines, an institution of society, and a manner of practice. It has increasing number and variety of specializes domains and demands of its practitioners [and] increasing array of skills and expertise.

Reference: Public Health Agency of Canada. Core Competencies for Public Health in Canada. Release 1.0, 1-25. 2007. Ottawa, Public Health Agency of Canada. p.13

*Collaboration* is defined as:

A recognized relationship among different sectors or groups, which have been formed to take action on an issue in a way that is more effective or sustainable than might be achieved by [any one group or sector] acting alone.

Reference: Public Health Agency of Canada. Core Competencies for Public Health in Canada. Release 1.0, 1-25. 2007. Ottawa, Public Health Agency of Canada. p.9

**Building and Maintaining Collaborations**

1. Tell me about your experience/s with collaborations between primary care and public health. (Consider the question from the context of your role, such as a policy maker, manager, front line worker.)
2. Why do you think some collaborations between primary care and public health have worked while others have not?

**Prompts:** **At a systems level,** What fosters/limits building and/or maintaining such collaborations? (social, economic, political, health environments, policies; funding structures; legislation.) **At an organizational level** what fosters/limits building and/or maintaining such collaborations? (organizational mandates, leadership, funding, program delivery models.) **At an interpersonal level** what fosters/limits building and/or maintaining such collaborations? (Team function and structure, roles, personal connections to colleagues, working styles.)

1. When do you think collaboration between primary care and public health is not desirable? Is there a time and place when it is not a good way to go? Is there a time and place when it is a good way to go?

***Prompts****:* Take into account different populations/ conditions/ circumstances where collaboration/s may be more or less desirable?

1. Tell me about any unintended or surprising consequences or harmful effects of collaborations between primary care and public health that you have experienced. Why do you think these things happened?
2. Describe what a successful collaboration between primary care and public health looks like.

***Prompts:*** Consider ***p***rocesses such as communication patterns, leadership styles.

1. How would you know that it was successful?

***Prompts***: What difference does a successful collaboration make? What positive outcomes have you seen? Consider health outcomes and/or health service delivery improvements. Were there changes in the quality of worklife, better retention or recruitment of staff or improved continuity of care?

1. Given what you have told me, in general what do you think is **essential** to have in place to grow a successful primary care and public health collaboration?

**Other**

1. Is there anything else that you think we should know about primary care and public health collaboration?

The research team may want to follow up with you at a later date, either by phone or email. We may want to check our interpretation of the data, or collect more information from you related to this study or to the larger program of research about collaborations between primary care and public health. **Do you agree to allow us to contact you again at a later date for such purposes?**

Yes ____ Thank you.

No ____ Thank you. We will not contact you again.

If yes, enter name and contact information (email or phone number) for follow up at a later date if required.

**Name: ___________________________________________**

**Contact: _________________________________________**

As we mentioned when we first contacted you, we are interested in talking to many front line workers, policy makers and decision-makers (e.g., managers) who have been involved with collaborations between public health and primary care.

Perhaps you have had a chance to think about people who you think would be appropriate for us to interview. We are interested in people who work in Ontario, BC, Nova Scotia at either a local, regional or provincial level or key informants at a national level.

**Please suggest anyone whom you think we should interview**

Name:

Contact Information:

Including some basic demographics (Area of work primary care of public health and health discipline; How would you describe this person?)

Name:

Contact Information:

Including some basic demographics (Area of work primary care of public health and health discipline; How would you describe this person?)

Name:

Contact Information:

Including some basic demographics (Area of work primary care of public health and health discipline; How would you describe this person?)

Name:

Contact Information:

Including some basic demographics (Area of work primary care of public health and health discipline; How would you describe this person?)

**Thank you for your participation. Your input has been very valuable.**
